# Knowledge and Preventive Practices Toward COVID-19 Among Sex Workers in Chiang Mai, Thailand

**DOI:** 10.3390/ijerph22121814

**Published:** 2025-12-03

**Authors:** Sameen Ashfaq, Kriengkrai Srithanaviboonchai, Patumrat Sripan, Arunrat Tangmunkongvorakul, Natthapol Kosashunhanan

**Affiliations:** 1 Research Institute for Health Sciences, Chiang Mai University, Chiang Mai 50200, Thailand; sameen_ashfaq@cmu.ac.th (S.A.); kriengkrai.s@cmu.ac.th (K.S.); patumrat.sripan@cmu.ac.th (P.S.); arunrat.tang@cmu.ac.th (A.T.); 2 Faculty of Medicine, Chiang Mai University, Chiang Mai 50200, Thailand

**Keywords:** COVID-19, knowledge, health behaviors, sex workers, Chiang Mai, Thailand

## Abstract

Sex workers were disproportionately affected by the COVID-19 pandemic due to precarious working conditions. This cross-sectional study was conducted in 2022 among 264 sex workers in Chiang Mai, Thailand, during the transition to the endemic phase, to evaluate their COVID-19 knowledge and preventive practices. Face-to-face interviews were used. Descriptive statistics were used to describe sample characteristics. Factors associated with knowledge and preventive practices were identified using the Mann–Whitney U test or Kruskal–Wallis test as appropriate. Independent factors associated with preventive practices were assessed through linear regression. The median scores for knowledge and preventive practices were 10 (interquartile range (IQR) = 9–10) and 5 (IQR = 3–5), respectively. In univariate analysis, females scored higher in knowledge than males. For preventive practices, females vs. males, older vs. younger, heterosexual vs. homosexual/bisexual, longer vs. shorter career, worked in massage parlors vs. pubs/bars, and having child vs. none showed higher rates. In multivariate analysis, being male (*β* = −1.87; 95%CI; −0.87 to −0.88) and single (*β* = −1.15; 95%CI; −2.28 to −0.02) were independent predictors of lower rates of preventive practices. Despite having good knowledge, certain groups of sex workers’ COVID-19 preventive behaviors remain inadequate, emphasizing the need for targeted interventions to enhance pandemic preparedness.

## 1. Introduction

Coronavirus disease 2019 (COVID-19) rapidly spread worldwide and escalated into a global pandemic after the initial cases were reported in Wuhan, China, in December 2019. The World Health Organization (WHO) declared COVID-19 a global health pandemic on 11 March 2020, and called for collaborative efforts from all countries to prevent the rapid spread of COVID-19 [1]. On 14 January 2020, Thailand detected its first laboratory-confirmed case of COVID-19 [2], becoming the first country outside of China to report such an occurrence. This initial case was the precursor to a series of sporadic imported cases and subsequent local transmissions. In response to emerging clusters, the Thai authorities shut down entertainment venues, pubs, and sporting arenas by mid-March. The situation intensified by early April when the government declared a state of emergency, instituted a curfew, and imposed stringent travel restrictions, including halting all commercial international flights. These measures, which varied in intensity and scope, were part of a broader strategy to effectively mitigate the spread of COVID-19 across the nation.

Thailand ranks eighth globally in terms of sex work, with approximately 250,000 individuals working in the industry, despite its illegal status [3]. Female sex work is more visible and recognized, largely due to its connection to tourism. Most women work in venues such as bars, massage parlors, and brothels, while some are involved in street-based sex work. In contrast, male sex work is less visible and generally faces a greater stigma. Male sex workers may work with both male and female clients, either independently or as part of an agency or network.

Among the most vulnerable groups affected by COVID-19 preventive measures are sex workers [4,5,6,7,8,9]. An online survey conducted in April and May 2020 gathered responses from over 20,000 lesbian, gay, bisexual, transgender, and intersex (LGBTI) individuals in 138 countries and revealed that 1% of respondents began performing sex work and that 2% continued selling sex during the COVID-19 pandemic, putting themselves at risk of exposure to the virus [10]. A global survey [11] across 55 countries revealed that during the COVID-19 pandemic, sex workers struggled to access social protection and economic support available to the general population, including income and housing relief. In Thailand, the nationwide closure of the estimated more than 23,000 entertainment venues where sex workers work meant that most lost the ability to earn an income overnight [12]. A small number of sex workers who continued to work despite the lockdown did so with weak negotiating powers, increasing their risk of exposure to COVID-19 and other infectious diseases [13].

Given their work in informal settings with limited access to essential services, this lack of support heightened their economic difficulties and vulnerability. This vulnerability is not limited to COVID-19 but might extend to other infectious diseases, accentuating the need for inclusive health policies that address the needs of all societal groups. Therefore, it is crucial for governments to implement inclusive responses that encompass all citizens, especially vulnerable groups such as sex workers, to ensure comprehensive public safety [14,15].

The health and well-being of sex workers are closely tied to those of their clients and the wider community [16]. Understanding epidemiological dynamics and transmission pathways is essential, as they significantly influence sex workers’ practices in disease transmission. Consequently, it is crucial to equip sex workers with accurate information and education to prevent the spread of diseases, including COVID-19, but also to ensure that they are not forced to continue sex work merely for survival.

Focusing on the knowledge and preventive practices of sex workers is essential for understanding their role in managing infection risk, particularly in the context of infectious disease outbreaks such as COVID-19 [17,18]. Notably, there is a significant lack of information regarding their knowledge of and preventive practices against COVID-19. This lack of data hampers the ability to comprehend fully how the pandemic affects sex workers. As such, a study is needed to address these gaps by evaluating the knowledge and preventive practices of sex workers in relation to COVID-19.

This study was carried out during the adaptation to the endemic phase of COVID-19 in Chiang Mai, Thailand, a region known for its tourism and sex industry, in 2022. General public health measures which include hygiene practices, mask mandates, and social distancing were still recommended. Vaccinations were widely adopted during this period. There was no specific health guideline for sex workers.

Studying the knowledge of COVID-19 and preventive measures among sex workers offers crucial public health insights. These workers typically have close physical interactions with clients, find it difficult to wear masks, and often face stigma that makes them hesitant to seek healthcare. Furthermore, the criminalization of sex work can discourage individuals from accessing health services due to concerns about potential legal repercussions. The results of this study could help formulate policies that enhance health protection for this at-risk group during future epidemics. The findings of this study are especially relevant given the ongoing global health situation, where the emergence of new virus strains, along with fluctuating case numbers, highlights the necessity for ongoing vigilance and flexible strategies, ensuring that marginalized communities such as sex workers are not only included but also adequately protected in the global pandemic response [19].

## 2. Methods

### 2.1. Study Setting and Study Population

This study adopted a cross-sectional analytic design, which is most effective for evaluating health conditions and their determinants in hard-to-reach populations like sex workers. This study was conducted in Chiang Mai city, Northern Thailand, from March to October 2022. This period corresponded with the transition to the endemic phase. This study was part of a larger investigation into the health and health behaviors of sex workers in cities. The research team accessed potential participants through established networks of local government and nongovernmental organizations (NGOs) that were already familiar with and trusted by sex worker communities. All sex workers who visited the clinics of these organizations during the specified period were invited to express their interest in participating in this study. The eligibility criteria included individuals who identified themselves as sex workers, were 18 years of age or older, and had engaged in sexual activity within the past 12 months.

### 2.2. Measures

The questionnaire consisted of 3 sections: sociodemographic (11 items covering age, gender, education, marital status, sexual identity, career duration, workplace, biological kids, family members, accommodation, and monthly income), knowledge of COVID-19 (10 items), and preventive practices (10 items) (File S1 in Supplementary Materials). These sections were designed to capture comprehensive data relevant to the sex worker population in Chiang Mai.

A set of questions assessing COVID-19 knowledge was adapted from established research tools [20,21] to evaluate multiple key areas. These areas include risk factors, susceptibility, symptoms, the incubation period, disease severity and asymptomatic transmission, treatment options, isolation and quarantine practices, preventive practices, and modes of transmission. The participants earned 1 point for each correct response, whereas incorrect or ‘do not know’ answers were given 0 points. The maximum achievable score was 10 points.

A set of questions assessing COVID-19 preventive practices was also adapted from existing research tools [20,21]. Preventive practices were evaluated through questions addressing mask-wearing, hand hygiene, physical distancing, respiratory hygiene, limiting social interactions, seeking medical advice, disinfecting the home environment, and avoiding close contact. Responses were scored on a frequency scale, with ‘always’ receiving 1 point and ‘never’ or ‘sometimes’ receiving 0 points. The maximum possible score for preventive practices was 10 points.

### 2.3. Data Collection

During the data collection period, some bars, clubs, and similar venues had been opened. Generally, sexual encounters took place in the same venues. This study recruited participants over 18 years of age who had engaged in sex work in the past year. NGO staff contacted them at workplaces or the NGO clinic to schedule appointments. A pre-interview screening ensured eligibility, followed by informed consent. To maintain confidentiality, interviews were conducted in private rooms with only research staff present, and participants from the same venue were grouped together.

The one-on-one interviews were conducted by trained interviewers. The training emphasized the use of interview tools and the administration of questionnaires. Each interview lasted approximately 15–20 min, with data being collected via a computer-assisted questionnaire using portable tablets. All the data were securely recorded via the Research Electronic Data Capture (REDCap version 11.4.4) platform.

### 2.4. Statistical Analysis

The data were summarized via descriptive statistics. The knowledge score was not normally distributed. The sample size was not calculated considering some multiple category variables. We therefore used non-parametric statistics to identify factors associated with knowledge and practice scores, the Mann–Whitney U test for two independent groups and the Kruskal–Wallis test for comparisons involving more than two independent groups. As the practice score was normally distributed, factors influencing practice were identified through univariable and multivariable linear regression. A cut-off value of ≥80% was set to define good knowledge and sufficient practice on the basis of a review of similar studies. Statistical significance was set at *p* < 0.05. Spearman’s rank correlation was used to examine the relationship between knowledge and preventive practices [22].

### 2.5. Ethical Considerations

This study was approved by the Human Experimentation Committee of the Research Institute for Health Sciences, Chiang Mai University. All methods were performed in accordance with relevant guidelines and regulations, including the Declaration of Helsinki. The participants were informed about this study’s objectives, and their anonymity and confidentiality were safeguarded. All the participants provided informed consent and received THB 300 (approximately USD 9) for their participation in the interviews.

## 3. Results

Among the 264 participants, 52.3% were male and 47.7% were female. The demographic profile revealed a predominantly young population aged 20 to 40, with 68.2% being single and having a diverse range of educational backgrounds. A notable number were employed in bars (26.5%) and traditional massage parlors (25%), highlighting these as common venues for sex work. The income distribution suggests a significant portion earned a moderate income (THB 10,000–20,000/month, 34.8%), with a small percentage earning higher amounts (THB > 20,000/month, 27.3%) (Table 1).

Over 80% of the participants correctly answered all but one of the COVID-19 knowledge questions. The exception was question K10, where 26.1% mistakenly believed there was an effective cure for COVID-19, resulting in fewer than 80% correct responses. In contrast, the highest percentage of correct answers (97.3%) was for question K1, which addressed susceptibility to infection. More detailed results can be found in Table 2.

With respect to COVID-19 preventive practices, the most common practice was always wearing a mask when going out (P1), with 79.9% adherence, followed by respiratory hygiene (P2), with 76.5% adherence. In contrast, avoiding contact with contaminated surfaces (P10) was the least common practice, with only 33.3% adherence (see Table 3 for details).

The participants’ overall understanding of COVID-19 was reflected in a median knowledge score of 10 (IQR = 9–10), with 89% correctly answering at least eight questions and 55.7% achieving a perfect score of 10. The median score for COVID-19 preventive practices was 5 (IQR = 3–5), with only 32.2% demonstrating sufficient preventive practices (≥8). A weak positive correlation (ρ = 0.218, *p* < 0.001) was found between knowledge and practice scores.

Females scored higher in knowledge (median = 10, IQR = 9.0–10.0) than males did (median = 10, IQR = 8.0–10.0; *p* = 0.036). Knowledge increased with age, peaking among those aged 31–50 (median = 10, IQR = 9.0–10.0), although this trend was not statistically significant (*p* = 0.140). Career duration showed a similar trend, with participants having 2.1–10 years of experience scoring highest (median = 10, IQR = 9.0–10.0; *p* = 0.495). No significant differences were observed across education level, marital status, sexual identity, workplace, or accommodation type. Participants from smaller households (1–5 members) tended to score slightly higher (median = 10, IQR = 9.0–10.0) than those in larger households (≥6 members: median = 9.0, IQR = 7.0–9.5; *p* = 0.057).

Compared with their male counterparts, female sex workers adhered more to preventive practices (*p* < 0.001). Age significantly impacted preventive practice scores (*p* < 0.001), with older participants scoring higher, particularly those aged 41–50 years (median = 5.5, IQR = 4.0–10.0), than those under 20 years (median = 3.0, IQR = 1.5–6.0).

Sexual identity also influenced preventive practice scores (*p* < 0.001), with heterosexual participants scoring significantly higher (median = 6.5, IQR = 4.0–9.0) than homosexual (median = 3.5, IQR = 2.2–5.0) and bisexual participants (median = 4.0, IQR = 2.0–7.0). Career duration was another significant factor (*p* = 0.033), with longer career durations being associated with higher scores, as those with over 10 years of experience had a median score of 6.0 (IQR = 3.0–10.0) compared with 5.0 (IQR = 2.0–8.0) for those with less than 2 years of experience.

The type of workplace significantly impacted preventive practice scores, with participants working in pubs and bars (median = 3.5, IQR = 2.0–7.0) and massage parlors (median = 7.0, IQR = 4.0–10.0) showing notable differences (*p* = 0.008 and *p* = 0.009, respectively). Parenthood also played a role, with participants who were parents scoring higher (median = 6.0, IQR = 3.0–9.0) than non-parents did (median = 5.0, IQR = 2.0–8.0, *p* = 0.032).

No significant differences in preventive practice scores were observed across education level, marital status, family size, accommodation type, or income level (Table 4).

The regression analysis revealed that male sex and single marital status were significant predictors of lower rates of COVID-19 preventive practices in both the univariate and multivariate analyses (*p* < 0.05). Younger age groups (<20 years and 20–30 years), shorter career durations (0–2 years and 2.1–5 years) and non–heterosexual identities, and having biological kids were associated with lower preventive practices in the univariable analysis (*p* < 0.05) but were not significant in the multivariable model. Further details are presented in Table 5 and Table 6.

## 4. Discussion

The current study offers significant insights into the knowledge and preventive practices regarding COVID-19 among sex workers in Chiang Mai, Thailand. With a sample of 264 participants, the demographic profile reveals a predominantly male and younger age. The high level of formal education among participants and the majority being single suggest a demographic that may be more receptive to health education initiatives. Additionally, the representation of diverse sexual identities—49% identifying as bisexual, 45.1% as heterosexual, and 6.1% as homosexual—underscores the complexity of the community’s health needs and the potential for varied responses to health messaging.

The findings on knowledge and preventive practices among participants are particularly significant. The high median knowledge score of 10 (IQR = 9–10) indicates a strong understanding of COVID-19, with 89% of participants correctly answering at least eight knowledge questions. However, the concerning fact that 26.1% of participants mistakenly believed there is an effective cure for COVID-19 highlights a critical gap in understanding that needs to be addressed through targeted educational interventions. In contrast, the median score of 5 (IQR = 3–5) for preventive practices reflects a troubling lack of adherence to recommended measures, with only 32.2% of participants demonstrating sufficient preventive behaviors. This discrepancy between knowledge and practice is echoed in global studies. For example, a study in Ghana reported that while many individuals had a good understanding of COVID-19, adherence to preventive practices, such as mask-wearing and social distancing, was significantly lower [23]. Similarly, research conducted in Egypt indicated that while participants were well informed about COVID-19, they were not effectively implementing preventive measures [24]. These comparisons underline a common trend across various contexts where high levels of knowledge do not necessarily translate into effective preventive practices. This suggests the need for comprehensive strategies that not only educate individuals about COVID-19 but also address barriers to adherence, such as access to resources, social norms, and behavioral incentives. Targeted interventions, particularly in communities with significant misconceptions, are essential to bridge the gap between knowledge and actual health practices.

Gender emerged as an independent factor linked to knowledge and preventive practices [25]. In this study, females scored significantly higher than males in both knowledge and preventive practices. This finding aligns with previous research, which showed that females generally outperformed males in these areas [26]. One possible explanation is that women tend to be more proactive about their health, often seeking out information and engaging in preventive health behaviors, such as regular check-ups and vaccinations. This pattern indicates that gender-specific health promotion strategies might be essential for effectively engaging male sex workers and improving their protective behaviors.

Cohabitation and parenthood emerged as significant factors. This study revealed that being single was an independent predictor of lower scores in preventive practices. This aligns with findings from a large multinational study indicating that single individuals were less likely to engage in preventive health behaviors during the pandemic [27]. Additionally, parents scored higher in preventive practices compared to non-parents, implying that having children may encourage individuals to adopt safer behaviors. However, there are no studies that directly compare preventive practices—such as mask-wearing and social distancing—between parents and non-parents during COVID-19. The study results suggest that health interventions targeting this population could be more effective by highlighting the protective motivations linked to parenthood.

Participants with older age and longer career duration had higher preventive practices scores. This observation aligns with a study in India, which reported that older sex workers demonstrated greater knowledge and engagement in safer practices, likely due to their longer experience in the field and heightened awareness of health risks [28]. Additionally, a study in Africa reported that younger individuals tended to have lower adherence to preventive practices, attributed to a combination of factors including lower risk perception and less access to information [29]. Shorter career durations among workers in health-related fields have been associated with lower levels of knowledge and practice adherence, suggesting that experience plays a crucial role in shaping health behaviors [30]. These findings highlight the importance of considering demographic variables when designing public health interventions tailored for sex workers.

This study also revealed that sexual identity influenced preventive practice scores, showing that heterosexual participants scored significantly higher than bisexual and homosexual participants. These findings align with a study conducted in Sweden [31]. This difference may be attributed to the fact that heterosexual individuals typically encounter fewer barriers to accessing preventive services due to societal norms. In contrast, bisexual and homosexual individuals may face discrimination or stigma in healthcare settings, which can limit their access to essential preventive health resources. This disparity highlights the need for further exploration of the social and cultural factors that affect health behaviors across various sexual identities.

Workplace environment was another critical factor affecting preventive practices. Participants working in higher-risk settings, such as pubs and bars, demonstrated lower adherence to preventive behaviors compared to those in massage parlors. This mirrors findings from a scoping review of the literature on the impact of the COVID-19 pandemic on sex workers, where sex workers in informal venues reported lower use of protective measures due to harsher working conditions and lack of support [32]. This finding underscores the importance of implementing tailored workplace policies that promote safer practices in environments where sex work is conducted.

A key strength of this study is its focus on a marginalized, vulnerable, and hard-to-reach population often overlooked in public health research. The use of multivariate analysis allowed the identification of specific demographic and social factors influencing COVID-19 preventive practices, providing valuable insights for targeted interventions.

However, several limitations must be acknowledged. In this study, preventive practices were evaluated according to general daily behaviors that were not specifically linked to encounters in sex work. The cross-sectional nature of this study limits the ability to infer causality between knowledge and practice, as well as the hypothesized predictor and outcome variables. The reliance on self-reported data may introduce recall and reporting bias, particularly in sensitive areas such as sexual behavior and compliance with preventive practices. Participants might provide answers they believe were more socially acceptable or favorable, rather than their true feelings or behaviors. Moreover, this study was conducted several times after the peak of the pandemic, potentially limiting its relevance, as behaviors and knowledge evolved over time, which could lead to inaccuracies in the data. Future research should prioritize longitudinal studies to monitor these changes and assess the long-term effectiveness of public health interventions.

In addition, intervention studies that measure the direct impact of targeted health promotion strategies are essential to enhance the practical application of knowledge and improve health outcomes among vulnerable groups.

## 5. Conclusions

This study revealed a significant gap between COVID-19 knowledge and the adoption of preventive practices among sex workers in Chiang Mai. While knowledge levels are generally high, the practical application of preventive practices has remained insufficient. Multivariate analysis revealed that sex and age were significant predictors of preventive practices, with females and older individuals demonstrating better adherence. These findings underscore the need for public health interventions that target broader behavioral factors beyond demographic characteristics. Tailored approaches addressing gender-specific needs, empowering younger individuals, and considering workplace settings are critical for improving the implementation of preventive practices.

## Figures and Tables

**Table 1:** Demographic characteristics of the sex workers (*n* = 264).

Characteristics	*n* (%)
**Gender**	
Male	138 (52.3)
Female	126 (47.7)
***Age*** (Median = 31 years, IQR = 25–28)	
<20 years	13 (4.9)
20–30 years	114 (43.2)
31–40 years	96 (36.4)
41–50 years	41 (15.5)
***Education***	
Primary	55 (20.8)
Secondary	75 (28.4)
High school	65 (24.6)
College/university	24 (9.1)
Other ^α^	8 (3)
No formal education	37 (14.1)
***Marital status***	
Single	180 (68.2)
Have a partner	53 (20.1)
Separated/divorced/widowed	31 (11.7)
***Sexual identity***	
Heterosexual	119 (45.1)
Homosexual	16 (6.1)
Bisexual	129 (48.9)
***Career duration*** (Median = 4 years, IQR = 2–8)	
0–2 years	78 (29.5)
2.1–5 years	81 (30.7)
5.1–10 years	63 (23.9)
>10 years	42 (15.9)
**Workplace**	
Karaoke	37 (14)
Traditional massage	66 (25)
Spa and Sauna	13 (4.9)
Restaurants	4 (1.5)
Café	2 (0.8)
Rural roadside bar	4 (1.5)
Pub/bar	70 (26.5)
Massage parlor	44 (16.7)
Other ^β^	91 (34.5)
***Biological kids***	
Yes	127 (48.1)
No	137 (51.9)
***Family members***	
1 person (living alone)	106 (40.2)
2–3 persons	89 (33.7)
4–5 persons	60 (22.7)
≥6 persons	9 (3.4)
***Accommodation***	
House	71 (26.9)
Dormitory	46 (17.4)
Rent a room	139 (52.7)
Other ^δ^	8 (3)
***Monthly income* (THB)** (Median = 15,000, IQR = 10,000–25,000)	
≤5000	9 (3.4)
5001–10,000	91 (34.5)
10,001–15,000	51 (19.3)
15,001–20,000	41 (15.5)
>20,000	72 (27.3)

IQR = interquartile range, ^α^ diploma, vocational certificates, ^β^ sex worker networking app, ^δ^ workplace or friend/relative’s house.

**Table 2:** The number and percentage of sex workers who answered each COVID-19 knowledge question correctly (N = 264).

Knowledge Questions	Correct *n* (%)
**K1:** All population is generally susceptible to infection. (true)	257 (97.3%)
**K2:** The main clinical symptoms of COVID-19 are fever, fatigue, dry cough. (true)	255 (96.6%)
**K3:** The COVID-19 virus spreads via respiratory droplets of infected individuals. (true)	251 (95.1%)
**K4:** COVID-19 disease is highly infectious and spreads quickly. (true)	246 (93.2%)
**K5:** Hand-washing, mask-wearing, and social distancing effectively prevent COVID-19. (true)	246 (93.2%)
**K6:** The incubation time of the disease is 14 days, typically 7 days. (true)	242 (91.7%)
**K7:** Not everyone with COVID-19 experiences severe symptoms. Elderly, chronically ill, obese at higher risk. (true)	240 (90.9%)
**K8:** Asymptomatic patients can transfer the virus to others. (true)	236 (89.4%)
**K9:** Isolate and treat confirmed patients; quarantine suspected cases for 14 days. (true)	224 (84.8%)
**K10:** There is currently no effective cure for COVID-19. (true)	195 (73.9%)

**Table 3:** The number and percentage of sex worker participants who responded that they practiced preventive practices during the COVID-19 epidemic (N = 264).

Practice Questions	Yes *n* (%)
**P1:** Always wear a mask when going out.	211 (79.9%)
**P2:** Cover mouth/nose when coughing/sneezing and avoid touching face, nose, or mouth with hands.	202 (76.5%)
**P3:** Wash hands with water, soap, or alcohol-based hand sanitizer.	145 (54.9%)
**P4:** Appropriate exercise and rest properly.	143 (54.2%)
**P5:** Seek medical advice when symptoms such as fever and cough appear.	142 (53.8%)
**P6:** Always disinfect home environment.	138 (52.3%)
**P7:** Reduce visits to your friends or relatives.	114 (43.2%)
**P8:** Reduce visits to crowded places.	107 (40.5%)
**P9:** Keep a distance of at least 2 m from others.	104 (39.4%)
**P10:** Avoid contact with contaminated public surfaces like elevator buttons and stair railings.	88 (33.3%)

**Table 4:** Knowledge and preventive practice scores of sex workers toward COVID-19.

Characteristics	Knowledge Score			Practice Score		
	Median	IQR	*p*	Median	IQR	*p*
**GENDER ^a^**			0.036 *			<0.001 *
Male	10.0	8.0–10.0		4.0	1.0–6.0	
Female	10.0	9.0–10.0		7.0	4.0–10.0	
**Age ^b^**			0.140			<0.001 *
<20 years	9.0	8.5–10.0		3.0	1.5–6.0	
20–30 years	10.0	8.0–10.0		4.0	2.0–7.0	
31–40 years	10.0	9.0–10.0		6.0	3.0–9.0	
41–50 years	10.0	9.0–10.0		5.5	4.0–10.0	
**Education ^b^**			0.282			0.888
Primary	10.0	9.0–10.0		5.0	2.0–5.0	
Secondary	10.0	9.0–10.0		5.0	2.0–5.0	
High school	10.0	9.0–10.0		5.0	3.0–5.0	
College/university	9.0	8.0–10.0		4.5	3.0–4.0	
Other ^α^	10.0	7.7–10.0		4.5	3.0–4.0	
**Marital status ^b^**			0.291			0.060
Single	10.0	9.0–10.0		5.0	2.0–8.0	
Have a partner	10.0	9.0–10.0		6.0	3.0–9.0	
Separated/divorced/widowed	10.0	9.0–10.0		6.0	3.0–9.0	
**Sexual identity ^b^**			0.483			<0.001 *
Heterosexual	10.0	9.0–10.0		6.5	4.0–9.0	
Homosexual	10.0	8.2–10.0		3.5	2.2–5.0	
Bisexual	10.0	8.0–10.0		4.0	2.0–7.0	
**Career duration ^b^**			0.495			0.033 *
0–2 years	9.0	8.0–10.0		5.0	2.0–8.0	
2.1–5 years	10.0	9.0–10.0		5.0	2.5–7.0	
5.1–10 years	10.0	9.0–10.0		6.0	3.0–9.0	
>10 years	10.0	9.0–10.0		6.0	3.0–10.0	
**Workplace ^a^**						
Karaoke ^a^	10.0	9.0–10.0	0.239	6.5	3.2–10.0	0.134
Traditional massage ^a^	10.0	9.0–10.0	0.707	5.0	3.0–9.0	0.730
Spa and sauna ^a^	9.5	8.0–10.0	0.811	7.0	2.0–10.0	0.261
Restaurants ^a^	10.0	9.2–10.0	0.340	6.5	4.2–8.7	0.445
Café ^a^	9.5	9.0–10.0	0.889	5.0	8.0–3.0	0.963
Rural roadside bar ^a^	9.5	7.5–10.0	0.778	5.5	2.5–8.5	0.929
Pub/bar ^a^	9.5	9.0–10.0	0.640	3.5	2.0–7.0	0.008 *
Massage parlor ^a^	10.0	9.0–10.0	0.213	7.0	4.0–10.0	0.009 *
Other ^βa^	10.0	9.0–10.0	0.995	5.0	3.0–8.0	0.993
**Biological kids ^a^**			0.127			0.032 *
Yes	10.0	9.0–10.0		6.0	3.0–9.0	
No	10.0	8.0–10.0		5.0	2.0–8.0	
**Family members ^b^**			0.057			0.638
1 person (living alone)	10.0	9.0–10.0		5.0	3.0–9.0	
2–3 persons	10.0	9.0–10.0		5.0	3.0–8.0	
4–5 persons	10.0	9.0–10.0		5.0	3.0–8.0	
≥6 persons	9.0	7.0–9.5		4.0	2.0–5.0	
**Accommodation ^b^**			0.440			0.221
House	10.0	9.0–10.0		5.0	3.0–8.0	
Dormitory	10.0	9.0–10.0		7.0	3.0–9.5	
Rent a room	10.0	9.0–10.0		4.0	2.5–9.0	
Other ^δ^	10.0	6.2–10.0		3.0	1.2–7.2	
**Monthly income (THB) ^b^**			0.112			0.057
≤5000	9.5	9.0–10.0		4.5	3.0–6.5	
5001–10,000	10.0	8.0–10.0		5.0	2.2–9.0	
10,001–15,000	10.0	9.0–10.0		4.0	2.0–7.2	
15,001–20,000	10.0	9.0–10.0		5.5	3.0–8.0	
>20,000	10.0	9.0–10.0		6.0	3.0–9.0	

* *p* < 0.05, ^α^ diploma, vocational certificates, ^β^ sex worker networking app, ^δ^ workplace or friend/relative’s house, ^a^ Mann–Whitney U test, ^b^ Kruskal–Wallis test, IQR = interquartile range.

**Table 5:** Factors associated with COVID-19 prevention practices via univariable regression analysis.

Characteristics	Univariable			
	95% CI			
	B	Lower	Upper	*p*
**Biological gender**				
Male	−2.20	−2.94	−1.46	<0.001 *
**Age (ref > 40 years)**				
<20 years	−2.77	−4.74	−0.80	0.006 *
20–30 years	−1.90	−3.03	−0.78	0.001 *
31–40 years	−0.62	−1.77	0.53	0.291
**Having biological kids**				
	0.856	0.078	1.634	0.031 *
**No. of family members (ref > 5 persons)**				
1 person	1.49	−0.73	3.70	0.190
2–3 persons	1.43	−0.81	3.66	0.210
4–5 persons	1.38	−0.90	3.66	0.240
**Accommodation (ref house)**				
Dormitory	0.12	−1.08	1.32	0.850
Rent a room	−0.58	−1.50	0.35	0.220
Other ^α^	−1.87	−4.24	0.50	0.120
**Marital status (ref having partner)**				
Single	−1.06	−2.05	−0.07	0.036 *
Separated/divorced/widowed	−0.15	−1.58	1.28	0.839
**Income (THB)**				
More than 10,000	−2.41	−1049	0.567	0.558
**Education (ref basic)**				
Advanced	−2.18	−1.43	1.00	0.724
**Sexual identity (ref heterosexual)**				
Homosexual	−2.60	−4.23	−0.97	0.002 *
Bisexual	−1.74	−2.51	−0.96	0.000 *
**Career duration (ref > 10 years)**				
0–2 years	−1.60	−2.80	−0.40	0.009 *
2.1–5 years	−1.20	−2.40	−0.01	0.049 *
5.1–10 years	−0.39	−1.64	0.86	0.541

* *p* < 0.05, B = regression coefficient, CI = confidence interval, basic (primary, secondary, high school), advanced (university, diploma, vocational certificate), ^α^ workplace or friend/relative’s house.

**Table 6:** Factors associated with COVID-19 prevention practices via multivariable regression analysis.

Characteristics	Multivariable			
	95% CI			
	aB	Lower	Upper	*p*
**Biological gender**				
Male	−1.87	−2.87	−0.88	<0.001 *
**Age (ref > 40 years)**				
<20 years	−1.37	−3.48	0.74	0.204
20–30 years	−0.55	−1.88	0.78	0.413
31–40 years	−0.23	−1.47	1.00	0.709
**Having biological kids**				
	−0.35	−1.36	0.66	0.494
**No. of family members (ref > 5 persons)**				
1 person	1.90	−0.50	4.30	0.120
2–3 persons	1.57	−0.69	3.83	0.170
4–5 persons	1.10	−1.18	3.37	0.340
**Accommodation (ref house)**				
Dormitory	−0.27	−1.61	1.07	0.694
Rent a room	−1.18	−2.37	0.01	0.051
Other ^α^	−1.78	−4.12	0.56	0.135
**Marital status (ref having partner)**				
Single	−1.15	−2.28	−0.02	0.046 *
Separated/divorced/widowed	−0.43	−1.90	1.04	0.563
**Income (THB)**				
More than 10,000	−0.56	−1.45	0.32	0.494
**Education (ref basic)**				
Advanced	−0.05	−1.24	1.13	0.929

* *p* < 0.05, B = regression coefficient, CI = confidence interval, basic (primary, secondary, high school), advanced (university, diploma, vocational certificate), ^α^ workplace or friend/relative’s house.

## Data Availability

The data presented in this study are available on request from the corresponding author.

## Supplementary Materials

The following supporting information can be downloaded at: https://www.mdpi.com/article/10.3390/ijerph22121814/s1, File S1: Demographic Data and Questionnaires for Knowledge and Preventive Practices on COVID-19.
