# Supplementary material for: Knowledge and Preventive Practices Toward COVID-19 Among Sex Workers in Chiang Mai, Thailand
Source: Int J Environ Res Public Health. 2025 Dec 3;22(12):1814. doi: 10.3390/ijerph22121814 (PMC12733356; doi:10.3390/ijerph22121814)
Supplement: Supplementary file 1 [file ijerph-22-01814-s001.zip › ijerph-3926943-supplementary.pdf]

# Interview form

## Part 1 demographic data

Participant ID F / M -☐☐-☐☐☐

Interviewer code ...............................................

Date of interview (dd-mm-yy Buddhist Era.) ☐☐-☐☐-☐☐☐☐

**Instruction** : Information received from the interview will be kept strictly confidential, please be comfortable and answer to these questions completely and as accurately as you can. If you have any questions or inquires, you can ask the interviewer any time.

<u>Section 1</u>**: Socio-demographic information**

1.1 What is your gender?

☐ 1. Male ☐ 2. Female

1.2 How old are you now?

.............................. Years old.

1.3 Where were you born?

☐ 1. Myanmar

☐ 2. Laos

☐ 3. Thailand

☐ 4. Other (Specify) ______________

1.4 What is your race/ethnicity?

☐ 1. Shan/Tai-Yai ☐ 2. Burmese

☐ 3. Laotian ☐ 4. Cambodian

☐ 5. Chinese ☐ 6. Thai

☐ 7. Other (Specify) ______________

1.5 Have you attended school ?

☐ 1. Yes, for ☐☐ years

☐ 2. No *(if **NO**, skip to **Q1.6**)*

1.5.1 In what education system?

☐ 1.Non-formal education system

☐ 2. Formal education system

1.5.2 What's your educational background?

☐ 1. Primary

☐ 2. Secondary

☐ 3. High school

☐ 4. College/university

☐ 5. Other, (specify) ______________

1.6 What is your religion?

☐ 1. Buddhism ☐ 2. Christianity

☐ 3. Islam ☐ 4. Other (Specify) ______________

1.7 What is your current work place? **(You can choose more than one answer)**

○ 1. karaoke ○ 2. Traditional Massage

○ 3. Spa & Sauna ○ 4. restaurants

○ 5. cafe ○ 6. Rural road-side bar

○ 7. Pub/ Bar ○ 8. Massage Parlor

○ 9. Other (Specify) _________

1.8 What is your current occupation?

☐ 1. Business owner (employee(s))

☐ 2. Freelance (part time)

☐ 3. Labourer / Employed for wages (full time)

☐ 4. Other (Specify) ______________

1.9 How long have you been in Thailand? I've been in Thailand for ............. months or .............years

1.10 Do you have ID card for staying in Thailand?

☐ 1. Yes

☐ 2. No *(if **No**, skip to **Q1.11**)*

If yes, type of card is

☐ 1.Passport ☐ 2.Pink card

☐ 3.Non-Thai identification card ☐ 4. Thai National ID Card

☐ 5.Other (Specify) ______________

1.11 Do you have a work permit or document registered in Thailand?

☐ 1.No ☐ 2.Yes

☐ 3. Thai National ID Card

1.12 What health insurance do you have?

☐ 1. Non Thai resident with health insurance ☐ 2. 30-baht health care

☐ 3. Social security ☐ 5. Don't have health insurance/ Self-pay ☐ 6. Don't know/Not sure ☐ 7. Other (Specify) ______________

1.13 What is your marital status?

☐ 1. Single

☐ 2. Have a partner

☐ 3. Separated/ Divorced/ Widowed

1.14 Do you have kid? **(biological kid only)**

☐ 1. Yes, (Specify) .........................persons

☐ 2. No

1.15 How many family members do you currently live with? ............................... persons

1.15.1Accommodation

☐ 1. House ☐ 3. Dormitory

☐ 2. Rent a room ☐ 4. Other, (specify) ......................

1.16 How much is your current income? Monthly income_____________Baht

1.17 What is the answer below that best describe your current household income when comparing with your household expenses?

☐ 1.Sufficient, with savings

☐ 2.Sufficient, with no savings

☐ 3.Insufficient / not enough to spend

## Part 2 : Questionnaire related to Knowledge towards COVID-19

| Sr.no | Questions | True | False | I don't know |
|---|---|---|---|---|
| 3.1 | All population is generally susceptible to infection. | | | |
| 3.2 | The main clinical symptoms of COVID-19 are fever, fatigue, dry cough. | | | |
| 3.3 | The COVID-19 virus spreads via respiratory droplets of infected individuals. | | | |
| 3.4 | COVID-19 disease is highly infectious and spreads quickly. | | | |
| 3.5 | Hand washing, wearing masks, and physical distancing are effective way to prevent the COVID-19 disease. | | | |
| 3.6 | The incubation time of the disease is 1–14 days, typically 7 days. | | | |
| 3.7 | Not all persons with COVID-19 will develop to severe cases. Only those who are elderly, have chronic illnesses, and are obese are more likely to be severe cases. | | | |
| 3.8 | Asymptomatic patients can transfer the virus to others. | | | |
| 3.9 | Confirmed patients should be isolated and treated in designated hospitals with effective isolation and protective conditions, and suspected cases should be in quarantine for 14 days. | | | |
| 3.10 | There is currently no effective cure for COVID-19. | | | |

## Part 3 : Questionnaire related to Practices towards COVID-19

| Sr.no | Questions | True | False | I don't know |
|---|---|---|---|---|
| 4.1 | Always wear a mask when going out. | | | |
| 4.2 | Cover mouth and nose when coughing or sneezing and avoid touching face, nose, or mouth with hands. | | | |
| 4.3 | Wash hands with water, soap, or alcohol-based hand sanitizer. | | | |
| 4.4 | Appropriate exercise and rest properly. | | | |
| 4.5 | Seek medical advice when symptoms such as fever and cough appear. | | | |
| 4.6 | Always disinfect home environment. | | | |
| 4.7 | Reduce visit your friends, or relatives. | | | |
| 4.8 | Reduce visits to crowded places. | | | |
| 4.9 | Keep distances from others at least 2 meters. | | | |
| 4.10 | Avoid direct contact with public facilities that may be infected, such as elevator buttons and stair railings. | | | |
